# Relationship between Anthropometric Parameters and Sensory Processing in Typically Developing Brazilian Children with a Pediatric Feeding Disorder

**DOI:** 10.3390/nu13072253

**Published:** 2021-06-30

**Authors:** Patrícia Junqueira, Dyandra Loureiro Caron dos Santos, Mariana Célia Guerra Lebl, Maria Fernanda Cestari de Cesar, Carolina Antunes dos Santos Amaral, Thais Coelho Alves

**Affiliations:** 1 Children Development Institute, São Paulo 04537-040, Brazil; dyandra@institutoinfantil.com.br (D.L.C.d.S.); mariana@institutoinfantil.com.br (M.C.G.L.); mariafernanda@institutoinfantil.com.br (M.F.C.d.C.); carolina@institutoinfantil.com.br (C.A.d.S.A.); thais@institutoinfantil.com.br (T.C.A.); 2 Graduate of Speech, Language and Hearing Sciences Department, São Paulo State University-UNESP, Marília 17525-900, Brazil

**Keywords:** pediatric feeding and eating disorders, anthropometry, modalities, sensorial, pediatric feeding disorders, food refusal, food selectivity, feeding problems, sensory processing

## Abstract

In this study, we aimed to relate anthropometric parameters and sensory processing in typically developing Brazilian children diagnosed with a pediatric feeding disorder (PFD). This was a retrospective study of typically developing children with a PFD. Anthropometric data were collected and indices of weight-for-age, length/height-for-age, and body mass index-for-age (BMI-for-age) were analyzed as z-scores. Sensory profile data were collected for auditory, visual, tactile, vestibular, and oral sensory processing. We included 79 medical records of children with a PFD. There were no statistically significant (*p* > 0.05) relationships between the anthropometric variables (weight-, length/height-, or BMI-for-age) and the sensory variables (auditory, visual, tactile, vestibular, or oral sensory processing). In conclusion, we found no relationship between anthropometric parameters and sensory processing in the sample of typically developing Brazilian children diagnosed with a PFD under study.

## 1. Introduction

A pediatric feeding disorder (PFD) is identified when a child has impaired oral intake that is not age-appropriate, which can be associated with multiple causal factors including medical, nutritional, oral–sensory–motor, and/or psychosocial dysfunctions [1]. These dysfunctions can be due to medical causes, including gastrointestinal, cardiorespiratory, and/or neurological problems, or nutritional causes, including malnutrition or a deficiency or restriction of a specific nutrient. For children with an oral–sensory–motor dysfunction and a PFD, they require the adjustment and modifications of food textures in order to be able to eat, as well as either positioning strategies or the use of specific feeding utensils. In addition, these children may present psychosocial dysfunctions caused by food aversion behaviors at mealtime, which, according to Berlin et al. [2] and Murphy et al. [3], contribute to parental stress and the use of inadequate feeding strategies that could be avoided if parents were instructed earlier on [1,4].

The prevalence of PFD has not been determined exactly, but is estimated to be from 25% to 30% among typically developing children and approximately 80% among children with neurological problems. However, previous studies have indicated that the percentage is growing every year among typically developing children and in other populations such as autistic, premature, and hyperactive children with attention deficit disorder, among others [4,5,6]. This growing trend is likely due to a true increase in the prevalence and awareness of PFD. 

A child’s growth and development constitute a complex, multifactorial process that is also influenced by nutritional aspects. Children with a PFD may experience deficits in energy or nutritional intake associated with significant weight loss or growth failure, significant nutritional deficiency, dependence on enteral feeding or oral nutritional supplements, or marked interference with psychosocial functioning [7,8,9,10]. This may lead to a more vulnerable nutritional status which predisposes the child to disease, nutritional deficiencies, and/or metabolic disorders in the short, mid, and long-term, which is one of the main concerns of both parents and caregivers [10,11,12].

Recently, a study analyzed the association between food selectivity and child growth and concluded that although food and nutritional intake differed between groups with and without a feeding disorder, a consistent relationship between food selectivity and child growth could not be verified [13]. Another study sought to associate selective behavior and the impact on nutrition and development with alterations in sensory perceptions that are commonly present in children with a PFD. These studies suggested that the impression people get from the sensory properties of foods plays a very important role in the way they select their food and how much they eat [14,15,16].

Schaaf and Roley [17] defined sensory processing as the means by which the central nervous system detects, modulates, discriminates, integrates, and organizes sensory stimuli and the response to sensory input itself. Eight senses make up the sensory systems: tactile, vestibular, proprioceptive, olfactory, gustatory, visual, auditory, and interoceptive [18]. The integration of information allows us to use our bodies effectively in an environment. Therefore, changes in sensory processing can manifest as an inappropriate response to this mechanism and/or as a compromised organization of sensory information. These dysfunctions can potentially have detrimental effects on typically developing children by compromising their participation in daily routines and functional activities, such as eating [19]. 

Eating is a sensory act. The sensory properties of food (taste, smell, texture, color, and appearance) affect our choices, as well as how much we eat [15]. Studies of children with an altered sensory profile in other populations, such as children with autistic spectrum disorder, have shown that they may exhibit selective, avoidant, or preferential behavior toward certain foods based on their sensory characteristics or specific brands, and may develop problems such as weight loss, adding to the frustration of their parents and family [20,21,22,23].

Navarrete-Muñoz et al. [24] reported that the interest in exploring the relationship between eating and sensory processing is due to the fact that the eating process involves the integration of sensory domains which trigger individual responses to the food characteristics, and that early childhood is a period during which food preferences and/or aversions are established. 

More evidence is needed to understand the nature of these relationships, mainly in the population of typically developing children with a PFD. Therefore, the purpose of this study was to relate anthropometric parameters and the sensory profiles of typically developing Brazilian children with a PFD. The hypothesis of the study was that altered sensory processing in the study sample may be an interfering factor in the anthropometric data.

Notably, investigating the association between sensory processing disorders and nutritional status in typically developing children may shed light on how to prevent or minimize the harmful consequences to children’s health and contribute positively to the diagnosis and management of PFDs. 

## 2. Materials and Methods

### 2.1. Ethical Criteria

This study was approved by the Research Ethics Committee under protocol number 08120218.7.0000.5505 and followed all ethical criteria to be compliant with current legislation.

### 2.2. Design 

This was a retrospective clinical study of the medical records of typically developing children who received care at the Children Development Institute in São Paulo, Brazil, who were examined between 2018 and 2020.

### 2.3. Participants

For this study, we included and examined the medical records of typically developing Brazilian children of a high socio-economic profile with the characteristics of a PFD [1].

We applied the following selection criteria:

(A) Children who had shown an inappropriate oral intake of nutrients for their age for at least three months that was associated with one or more of the following items:
  - Medical dysfunction evidenced by one of the following:
    - Cardiorespiratory impairment during oral ingestion.
  - Nutritional dysfunction evidenced by one of the following:
    - Malnutrition;
    - Specific nutritional deficiency or significantly restricted intake of one or more nutrients resulting from a reduction in dietary variety.
  - Feeding skill dysfunction evidenced by one of the following:
    - Need to adapt the texture of liquids or food;
    - Certain position or equipment used to adapt food intake;
    - Use of strategies to adapt food intake.
  - Psychosocial dysfunction evidenced by one of the following:
    - Active or passive escape behavior when the child is eating or being fed;
    - Caregiver’s inappropriate management of the child’s food intake and/or nutritional needs;
    - Disrupted social functioning in the context of food;
    - Disrupted child–caregiver relationship associated with food.
(B) Absence of cognitive processes consistent with eating disorders.

Children with neurological disorders and children under seven months and over 36 months of age were excluded from the sample.

### 2.4. Dynamics of Care 

All participants in this study were treated at the same center dedicated to the care of children with feeding problems. This center is staffed by a group of professionals, including a nutritionist, occupational therapist, speech therapist, and psychologist. 

All of the medical records of the children included in this study covered the same protocol for the dynamics of care and contained data that were collected in an initial interview.

Complete anthropometric data and sensory profiles were available for all selected children.

### 2.5. Anthropometric Parameters

Prior to the first evaluation at the institute, parents were asked to provide important nutritional information including a detailed food record, food inventory, recent biochemical tests (when available), and updated growth charts extracted from medical records. We also collected weight-for-age, length/height-for-age, and BMI-for-age data. We entered the gathered anthropometric data into WHO Anthro software [25], which calculated the indices and plotted the results.

We used the graphs recommended by the World Health Organization (WHO) for boys and girls between the ages of 0–5 years [25]. The cutoff points used in the different curves are represented as z-scores, which indicate units of standard deviation from the median value (z-score of 0). 

For premature patients who were admitted to care at under two years of age and for extremely premature infants (gestational age < 28 weeks) admitted at under three years of age, the WHO curve was adjusted for chronological age. The cutoff points and nomenclature adopted for each z-score range followed the WHO recommendations [25]. We then classified the data as either age-appropriate or not age-appropriate.

### 2.6. Sensory Processing Profile Data

The occupational therapy team assessed the sensory profiles of typically developing children with feeding problems using the Infant Toddler Sensory Profile (ITSP) tool [26]. This tool consists of a questionnaire with forty-eight questions for the age group between seven and thirty-six months to be completed by the parents or primary caregiver of the baby or young child in order to collect information about the child’s sensory processing skills. For this study, the instrument was sent by email, along with instructions on how to properly complete the questionnaire, as described in the manual [27].

Parents rated the frequency of their child’s behavior on a scale from one (almost always) to five (almost never). The responses of the parents or caregiver were analyzed according to the standard instructions of the manual, and the sensory profile was scored based on the proposed score table [27]. After tabulating the scores, children were classified into profiles based on typical performance or probable/definite difference in performance for each type of sensory processing (auditory, visual, tactile, vestibular, and oral).

For greater objectivity, we opted to cross only the data from the sensory processing segments (auditory, visual, tactile, vestibular, and oral) and focus on the abilities of the senses and not on the neurological condition related to broader questions of sensory integration. We also provided a second classification by dichotomizing the data as either typical (typical performance) or atypical (probable difference or definite difference in performance) for each type of sensory processing [28].

### 2.7. Data Collection

We collected demographic data on the children by extracting data from the anthropometric assessment in their medical records and sensory profile analysis. The specific information for the analyses included weight (kg), weight-for-age anthropometric index (z-score), length/height (cm), length/height-for-age anthropometric index (z-score), BMI (kg/m²), BMI-for-age anthropometric index (z-score), auditory processing, visual processing, tactile processing, vestibular processing, and oral sensory processing.

### 2.8. Statistical Analysis

We employed descriptive statistics to analyze the data, which are presented as mean ± standard deviation when appropriate. For this study, we adopted a statistical significance level of *p* < 0.05. We tested the normality of the quantitative variables using a Shapiro–Wilk test, and found that the data were non-normally distributed. Therefore, we used nonparametric chi-squared tests.

## 3. Results

### 3.1. Study Sample

We included 79 medical records of children diagnosed with a PFD. The mean age in years was 1.80 ± 0.70 and the mean age at the onset of feeding problems was 7.06 ± 5.32 months. The data showed that 61 (82.4%) children were premature and 16 (21.6%) had food allergies, most frequently cow’s milk protein. The results showed that 55 (72.4%) children did not have family meals and that the same number required a distraction during meals.

### 3.2. Anthropometric and Sensory Processing Data

Our evaluation of the anthropometric parameters showed that the mean ± standard deviation for weight was 10.73 ± 2.50 kg; length/height was 82.43 ± 9.34 cm; and BMI was 15.53 ± 1.58 kg/m². The anthropometric indices showed that 68 (86.1%) and 74 (93.7%) children were in the appropriate classification in terms of weight-for-age and length/height-for-age, respectively, according to the z-score cutoff points. In the evaluation of the BMI-for-age anthropometric index, 66 (83.5%) children had a normal weight according to the z-score cutoff point (Table 1). Although we classified most of the anthropometric parameters as appropriate, there were a small number of children in the sample whose data in terms of weight, length/height, and BMI-for-age were classified below or even above what was appropriate for their age group.

With regard to sensory processing, we noted that 51 (64.6%) children showed typical performance in auditory processing, 48 (60.8%) in visual processing, 52 (65.8%) in tactile processing, and 43 (54.4%) in vestibular processing. However, for oral sensory processing, there was a higher frequency of children with a probable difference (32; 40.5%) and a definite difference in performance (31; 39.2%).

### 3.3. Relationship between Anthropometric Parameters and Sensory Profile

There were no statistically significant relationships between the weight-for-age anthropometric index (z-score) (Table 2) and visual (*p* = 0.745), auditory (*p* = 0.393), oral–sensory (*p* = 0.210), tactile (*p* = 0.709), or vestibular (*p* = 0.173) processing. In addition, there were no relationships between the length/height-for-age anthropometric index (z-score) (Table 3) and visual (*p* = 0.475), auditory (*p* = 0.552), oral–sensory (*p* = 0.605), tactile (*p* = 0.822), or vestibular (*p* = 0.463) processing. Finally, there were no differences between the BMI-for-age (Table 4) anthropometric index (z-score) and visual (*p* = 0.916), auditory (*p* = 0.067), oral–sensory (*p* = 0.431), tactile (*p* = 0.504), or vestibular (*p* = 0.303) processing.

Even when we dichotomized the anthropometric data (age-appropriate and not age-appropriate) and the sensory processing data (typical and atypical performance), in another type of analysis (Table 5), the results also revealed no significant relationships between the weight-for-age anthropometric index (z-score) and visual (*p* = 0.833), auditory (*p* = 0.197), oral–sensory (*p* = 0.321), tactile (*p* = 0.395), or vestibular (*p* = 0.189) processing. There were no significant relationships between the length/height-for-age anthropometric index (z-score) and visual (*p* = 0.326), auditory (*p* = 0.826), oral–sensory (*p* = 0.988), tactile (*p* = 0.777), or vestibular (*p* = 0.236) processing. Finally, there were no differences between the BMI-for-age anthropometric index (z-score) and visual (*p* = 0.494), auditory (*p* = 0.700), oral–sensory (*p* = 0.302), tactile (*p* = 0.102), or vestibular processing (*p* = 0.241).

## 4. Discussion

Exploring the relationship between anthropometric parameters and sensory processing in our sample was important because we believe that feeding behavior, which is influenced by a child’s sensory preferences and aversions to food at an early age, can compromise their interest and motivation to eat and may result in short, mid, and long-term health consequences. Understanding this relationship will help us detect PFD and discover more preventive and decisive treatments.

Our analysis of anthropometric parameters indicated that there were children with appropriate and inappropriate indices for their age group. It is important to highlight that clinical experience shows us that feeding problems are not always associated with malnutrition or growth deficiencies. In a study published by a Brazilian group from a center for child feeding disorders, most of the children were classified as normal weight, even though their developmental patterns tended to be in the lower percentiles, which was similar to our results [29].

In a recent comprehensive review of the nutritional aspects of children with feeding problems, which considered their relationship with anthropometric parameters, the authors observed that in some studies, children with feeding problem complaints had a significantly lower BMI than children without those complaints. In some of the studies reviewed, children with feeding disorders were less likely to be overweight or obese than those in the control group [13]. The findings for BMI-for-age partially agree with the results of our sample, since the findings for overweight subjects were numerically smaller than those for normal weight or underweight subjects in the analyzed population.

In other studies, children with feeding problems also presented with lower length/height-for-age index values than the control group [30,31]. This point deserves attention, since a short stature for a given age may signal a growth deficiency that might be related to chronic diseases such as food allergies, inflammatory bowel disease, and malnutrition [32], which are situations that can favor the development and/or maintenance of a PFD and long-term health complications [33]. We did not observe this statistically significant relationship in our results, although five (6.3%) children in our sample presented with short stature for their age. In this specific group, four (80%) had been diagnosed with a food allergy and/or gastrointestinal issues and were already undergoing appropriate treatment for such conditions.

Regarding the BMI-for-age parameter in our study, part of the assessed population proved to be overweight (*n* = 4; 5%), although this result was not statistically significant in terms of the sample. Children with food selectivity, even with a reduced dietary repertoire, may consume snacks and other foods/products with a high energy density and a poor nutritional value. Moreover, sensory issues can also affect the level of activity and perception of internal signs of hunger and satiety, thereby compromising the regulation of food intake and body weight [34,35,36].

Importantly, methodological issues in these studies should be highlighted, since several publications classified children with feeding problems from a parental and non-professional perspective. Furthermore, no single uniform method for classifying pediatric feeding disorders is available, which makes the population in question even more heterogeneous, so that it is not possible to establish reliable universal standards [13,37].

In this study, most children were classified as having an appropriate nutritional status based on the BMI-for-age index. This is a particularly important finding, since we observed in clinical practice that the diagnosis and referral of children with a PFD is often based on anthropometric assessments. Therefore, a clinical assessment based on anthropometric criteria may compromise the early diagnosis of PFD in children. We validated and reiterate the importance of anthropometric assessments. However, we emphasize that assessments of children should go beyond these parameters and include observations of the child’s mealtime behavior and their relationship with food and their family at mealtimes, as reported by Nogueira-de-Almeida [38]. In our sample, even a child with normal weight and a PFD could show compromised mealtime behavior. Some of these children can only consume a limited number of foods, do not participate in family meals, eat only as a distraction, eat at night, do not chew, present with a sensory aversion, etc., with biopsychosocial effects [1].

With regard to sensory processing, which has not been explored in previous studies of a typically developing population [28,39,40], we observed different profiles in the sample in this study. Clearly different profiles were most prevalent in the oral sensory processing segment of the data collected.

In our clinical practice and in agreement with other authors [41], children who present with changes in oral sensory processing usually have two distinct sensory patterns: (a) Inappropriate intraoral discrimination, which affects a child’s perception of food in the mouth, causing limited bolus formation, loss of food through the mouth and choking, or refusal of liquids and food textures that provide insufficient sensory input [41]; and (b) intraoral hypersensitivity, which interferes with the child’s proper interpretation of a stimulus and generates an exacerbated response in relation to the nature, intensity, and duration of the stimulus received, so that the child may choke (retching) depending on the type, texture, size, flavor, smell, temperature, viscosity, color, and/or appearance of the food, and limitations of the range of ingestion [41,42,43].

According to recent research, sensory processing dysfunctions can cause feeding and swallowing disorders such as food refusal and self-limited diets [42]. Although there is a lack of studies of typically developing populations, more research focusing on sensory processing by children with a PFD may help us understand certain mealtime behaviors that are still poorly understood in this specific population. 

The relationship between nutritional status and sensory processing has not been precisely determined in this population. Our results showed no statistically significant relationships between the weight-for-age (z-score), length/height-for-age (z-score), or BMI-for-age (z-score) anthropometric indices and sensory processing. Navarrete-Muñoz et al. [16] related BMI to changes in sensory processing in school-aged Spanish children between the ages of three and seven years. The authors did not find a positive association between increased BMI and altered sensory processing.

Other studies, such as those of Navarrete-Muñoz et al. [24], Moding et al. [44], and Suarez [45], reported that the effect of smell, texture, and taste on daily dietary choices directly affects feeding behavior and can have long-term effects, such as changes in body weight or BMI. These studies agree that changes in sensory processing may be related to low appetite, little interest in food-related activities, and less pleasure during eating.

In contrast to our results, Navarrete-Muñoz et al. [24] demonstrated that almost one third of their preschool and school-aged Spanish participants presented with atypical sensory performance and that a similar proportion of participants were overweight or obese. Although the associations between being overweight and the prevalence of atypical sensory results were not statistically significant in this population, the main findings indicated that an increase in BMI was significantly associated with a higher prevalence of atypical tactile sensitivity in children aged from three to seven years old. In a previously published [13] study with the same population, atypical performance for tactile sensitivity was significantly associated with lower adherence to the Mediterranean diet (that is, a low consumption of fruits, vegetables, grains, and cereals) which may be related to the high BMI-for-age results. This association between tactile sensitivity and specific food choices has been reported previously in the literature [42,46,47,48].

As for the limitations of this study, we collected data from medical records on a single date (beginning of follow-up care at the Institute) without a follow-up for our evaluation of the anthropometric parameters. Additionally, an evaluation of some measurements (e.g., body circumference and skin folds) that provide more information on body composition could have outlined a more specific relationship between the variables. With regard to sensory processing, it should be noted that the ITSP is not a diagnostic tool, but rather a screening measure completed by parents. Therefore, with the necessary indications, a comprehensive assessment is required to make a diagnosis through a clinical evaluation by an occupational therapist trained in sensory integration. In this respect, children classified according to ITSP scores may not necessarily present with typical or atypical sensory processing. Thus, an underestimation of our conclusions cannot be rejected. 

This study did not find a statistical relationship in our sample between the anthropometric parameters and the sensory profile of typically developing Brazilian children diagnosed with a PFD. 

## Figures and Tables

**Table 1:** Anthropometric and sensory processing characteristics of the sample.

	*n* (%)
**(z-score) Weight-for-age**	
Normal	68 (86.1%)
Underweight	9 (11.4%)
Overweight	2 (2.5%)
**(z-score) Length/Height-for-age**	
Normal	74 (93.7%)
Stunted	5 (6.3%)
**(z-score) BMI-for-age**	
Normal	66 (83.5%)
Wasted	9 (11.4%)
Overweight	4 (5.1%)
**Auditory Processing**	
Typical performance	51 (64.6%)
Probable difference	19 (24.1%)
Definite difference	9 (11.4%)
**Visual Processing**	
Typical performance	48 (60.8%)
Probable difference	28 (35.4%)
Definite difference	3 (3.8%)
**Tactile Processing**	
Typical performance	52 (65.8%)
Probable difference	18 (22.8%)
Definite difference	9 (11.4%)
**Vestibular Processing**	
Typical performance	43 (54.4%)
Probable difference	28 (35.4%)
Definite difference	8 (10.1%)
**Oral Sensory Processing**	
Typical performance	16 (20.3%)
Probable difference	32 (40.5%)
Definite difference	31 (39.2%)

**Table 2:** Relationship between weight-for-age anthropometric index (z-score) and sensory processing.

		Underweight *n* (%)	Normal *n* (%)	Overweight *n* (%)	Total *n* (%)	Chi-Squared Test	*p*-Value
**Visual Processing**	Typical performance	5 (55.6)	41 (60.3)	2 (100)	48 (60.8)	χ (4, 79) = 1.95, 0.745	0.745
	Probable difference	4 (44.4)	24 (35.3)	0 (0)	28 (35.4)		
	Definite difference	0 (0)	3 (4.4)	0 (0)	3 (3.8)		
**Auditory Processing**	Typical performance	8 (88.9)	42 (61.8)	1 (50)	51 (64.6)	χ (4, 79) = 4.10, 0.393	0.393
	Probable difference	0 (0)	18 (26.5)	1 (50)	19 (24.1)		
	Definite difference	1 (11.1)	8 (11.8)	0 (0)	9 (11.4)		
**Oral Sensory Processing**	Typical performance	1 (11.1)	15 (22.1)	0 (0)	16 (20.3)	χ (4, 79) = 5.85, 0.210	0.210
	Probable difference	6 (66.7)	26 (38.2)	0 (0)	32 (40.5)		
	Definite difference	2 (22.2)	27 (39.7)	2 (100)	31 (39.2)		
**Tactile Processing**	Typical performance	5 (55.6)	46 (67.6)	1 (50)	52 (65.8)	χ (4, 79) = 2.15, 0.709	0.709
	Probable difference	2 (22.2)	15 (22.1)	1 (50)	18 (22.8)		
	Definite difference	2 (22.2)	7 (10.3)	0 (0)	9 (11.4		
**Vestibular Processing**	Typical performance	7 (77.8)	35 (51.5)	1 (50)	43 (54.4)	χ (4, 79) = 6.37, 0.173	0.173
	Probable difference	2 (22.2)	26 (38.2)	0 (0)	28 (35.4)		
	Definite difference	0 (0)	7 (10.3)	1 (50)	8 (10.1)		

**Table 3:** Relationship between length/height-for-age anthropometric index (z-score) and sensory processing.

		Stunted *n* (%)	Normal *n* (%)	Total *n* (%)	Chi-Squared Test	*p*-Value
**Visual Processing**	Typical performance	2 (40.0)	46 (62.2)	48 (60.8)	χ (2, 79) = 1.49, 0.475	0.475
	Probable difference	3 (60.0)	25 (33.8)	28 (35.4)		
	Definite difference	0 (0)	3 (4.1)	3 (3.8)		
**Auditory Processing**	Typical performance	3 (60.0)	48 (64.9)	51 (64.6)	χ (2, 79) = 1.19, 0.552	0.552
	Probable difference	2 (40.0)	17 (23.0)	19 (24.1)		
	Definite difference	0 (0)	9 (12.2)	9 (11.4)		
**Oral Sensory Processing**	Typical performance	1 (20.0)	15 (20.3)	16 (20.3)	χ (2, 79) = 1.00 0.605	0.605
	Probable difference	3 (60.0)	29 (39.2)	32 (40.5)		
	Definite difference	1 (20.0)	30 (40.5)	31 (39.2)		
**Tactile Processing**	Typical performance	3 (60.0)	49 (66.2)	52 (65.8)	χ (2, 79) = 0.39, 0.822	0.822
	Probable difference	1 (20.0)	17 (23.0)	18 (22.8)		
	Definite difference	1 (20.0)	8 (10.8)	9 (11.4)		
**Vestibular Processing**	Typical performance	4 (80.0)	39 (52.7)	43 (54.4)	χ (2, 79) = 1.54, 0.463	0.463
	Probable difference	1 (20.0)	27 (36.5)	28 (35.4)		
	Definite difference	0 (0)	8 (10.8)	8 (10.1)		

**Table 4:** Relationship between BMI-for-age anthropometric index (z-score) and sensory processing.

		Wasted *n* (%)	Normal *n* (%)	Overweight *n* (%)	Total *n* (%)	Chi-Squared Test	*p*-Value
**Visual Processing**	Typical performance	6 (66.7)	39 (59.1)	3 (75)	48 (60.8)	χ (4, 79) = 0.96, 0.916	0.916
	Probable difference	3 (33.3)	24 (36.4)	1 (25)	28 (35.4)		
	Definite difference	0 (0.0)	3 (4.5)	0 (0.0)	3 (3.8)		
**Auditory Processing**	Typical performance	8 (88.9)	42 (63.6)	1 (25)	51 (64.6)	χ (4, 79) = 8.77, 0.067	0.067
	Probable difference	0 (0.0)	16 (24.2)	3 (75)	19 (24.1)		
	Definite difference	1 (11.1)	8 (12.1)	0 (0.0)	9 (11.4)		
**Oral Sensory Processing**	Typical performance	2 (22.2)	12 (18.2)	2 (50)	16 (20.3)	χ (4, 79) = 3.82, 0.431	0.431
	Probable difference	3 (33.3)	26 (39.4)	2 (50)	31 (39.2)		
	Definite difference	4 (44.4)	28 (42.4)	0 (0.0)	32 (40.5)		
**Tactile Processing**	Typical performance	4 (44.4)	46 (69.7)	2 (50)	52 (65.8)	χ (4, 79) = 3.33, 0.504	0.504
	Probable difference	3 (33.3)	14 (21.2)	1 (25)	18 (22.8)		
	Definite difference	2 (22.2)	6 (9.1)	1 (25)	9 (11.4)		
**Vestibular Processing**	Typical performance	5 (55.6)	34 (51.5)	4 (100)	43 (54.4)	χ (4, 79) = 4.85, 0.303	0.303
	Probable difference	4 (44.4)	24 (36.4)	0 (0.0)	28 (35.4)		
	Definite difference	0 (0.0)	8 (12.1)	0 (0.0)	8 (10.1)		

**Table 5:** Relationship between weight-for-age (z-score), length/height-for-age (z-score), and BMI-for-age (z-score) (all dichotomous) anthropometric indices and sensory processing (all dichotomous).

			Age-Appropriate *n* (%)	Not Age-Appropriate *n* (%)	Total *n* (%)	Chi-Squared Test	*p*-Value
**(z-score) Weight-for-age**	**Visual Processing**	Typical	41 (60.3)	7 (63.6)	48 (60.8)	χ (1, 79) = 0.04, 0.833	0.833
		Atypical	27 (39.7)	4 (36.4)	31 (39.2)		
	**Auditory Processing**	Typical	42 (61.8)	9 (81.8)	51 (64.6)	χ (1, 79) = 1.66, 0.197	0.197
		Atypical	26 (38.2)	2 (18.2)	28 (35.4)		
	**Oral Sensory Processing**	Typical	15 (22.1)	1 (9.1)	16 (20.3)	χ (1, 79) = 0.99, 0.321	0.321
		Atypical	53 (77.9)	10 (90.9)	63 (79.7)		
	**Tactile Processing**	Typical	46 (67.6)	6 (54.5)	52 (65.8)	χ (1, 79) = 0.72, 0.395	0.395
		Atypical	22 (32.4)	5 (45.5)	27 (34.2)		
	**Vestibular Processing**	Typical	35 (51.5)	8 (72.7)	43 (54.4)	χ (1, 79) = 1.72, 0.189	0.189
		Atypical	33 (48.5)	3 (27.3)	36 (45.6)		
**(z-score) Length/Height-for-age**	**Visual Processing**	Typical	46 (62.2)	2 (40.0)	48 (60.8)	χ (1, 79) = 0.96, 0.326	0.326
		Atypical	28 (37.8)	3 (60.0)	31 (39.2)		
	**Auditory Processing**	Typical	48 (64.9)	3 (60.0)	51 (64.6)	χ (1, 79) = 0.05, 0.826	0.826
		Atypical	26 (35.1)	2 (40.0)	28 (35.4)		
	**Oral Sensory Processing**	Typical	15 (20.3)	1 (20.0)	16 (20.3)	χ (1, 79) = 0.00, 0.988	0.988
		Atypical	59 (79.7)	4 (80.0)	63 (79.7)		
	**Tactile Processing**	Typical	49 (66.2)	3 (60.0)	52 (65.8)	χ (1, 79) = 0.08, 0.777	0.777
		Atypical	25 (33.8)	2 (40.0)	27 (34.2)		
	**Vestibular Processing**	Typical	39 (52.7)	4 (80.0)	43 (54.4)	χ (1, 79) = 1.41, 0.23	0.236
		Atypical	35 (47.3)	1 (20.0)	36 (45.6)		
**(z-score) BMI-for-age**	**Processing. Visual**	Typical	39 (59.1)	9 (69.2)	48 (60.8)	χ (1, 79) = 0.47, 0.494	0.494
		Atypical	27 (40.9)	4 (30.8)	31 (39.2)		
	**Auditory Processing**	Typical	42 (63.6)	9 (69.2)	51 (64.6)	χ (1, 79) = 0.15, 0.700	0.700
		Atypical	24 (36.4)	4 (30.8)	28 (35.4)		
	**Oral Sensory Processing**	Typical	12 (18.2)	4 (30.8)	16 (20.3)	χ (1, 79) = 1.07, 0.302	0.302
		Atypical	54 (81.8)	9 (69.2)	63 (79.7)		
	**Tactile Processing**	Typical	46 (69.7)	6 (46.2)	52 (65.8)	χ (1, 79) = 2.68, 0.102	0.102
		Atypical	20 (30.3)	7 (53.8)	27 (34.2)		
	**Vestibular Processing**	Typical	34 (51.5)	9 (69.2)	43 (54.4)	χ (1, 79) = 1.37, 0.241	0.241
		Atypical	32 (48.5)	4 (30.8)	36 (45.6)		
